# Retraction Note: Changes in the electron paramagnetic resonance spectra of albumin-associated spin-labeled stearic acid as a diagnostic parameter of colorectal cancer

**DOI:** 10.1186/s12957-016-0910-9

**Published:** 2016-06-02

**Authors:** Zhongchao Liu, Wenyi Zhang, Saijun Fan, Liang Wang, Ling Jiao

**Affiliations:** Department of Radiation Protection Center, Institute of Radiation Medicine, Chinese Academy of Medical Sciences & Peking Union Medical College, Tianjin Key Laboratory of Molecular Nuclear Medicine, 238 Baidi Road, Nankai District, Tianjin, 300192 People’s Republic of China

The authors are retracting this article [1] due to overlap with previous publications, most notably [2–4], without appropriate attribution. We apologize for any inconvenience caused.
